# COVID-19 vaccination program in the mainland of China: a subnational descriptive analysis on target population size and current progress

**DOI:** 10.1186/s40249-021-00909-1

**Published:** 2021-10-15

**Authors:** Wen Zheng, Xuemei Yan, Zeyao Zhao, Juan Yang, Hongjie Yu

**Affiliations:** 1 Shanghai Institute of Infectious Disease and Biosecurity, School of Public Health, Fudan University, Shanghai, China; 2 Key Laboratory of Public Health Safety, Ministry of Education, Fudan University, Shanghai, China; 3 Department of Infectious Diseases, Huashan Hospital, Fudan University, Shanghai, China

**Keywords:** Novel coronavirus disease 2019, Vaccination, Target population, China

## Abstract

**Background:**

China is facing substantial risks of imported coronavirus disease 2019 (COVID-19) cases and a domestic resurgence in the long run, and COVID-19 vaccination is expected to be the long-lasting solution to end the pandemic. We aim to estimate the size of the target population for COVID-19 vaccination at the provincial level in the mainland of China, and summarize the current progress of vaccination programs, which could support local governments in the timely determination and adjustment of vaccination policies and promotional measures.

**Methods:**

We conducted a descriptive study of the entire population in the mainland of China, between December 2020 and August 2021. By extracting provincial-stratified data from publicly available sources, we estimated the size of priority target groups for vaccination programs, and further characterized the ongoing vaccination program at the provincial level, including the total doses administered, the coverage rate, and the vaccination capacity needed to achieve the target coverage of 80% by the end of 2021. We used R (version 4.1.0) to complete the descriptive statistics.

**Results:**

The size of the target population shows large differences among provinces, ranging from 3.4 million to 108.4 million. As of 31 August, 2021, the speed of vaccine roll-out differs considerably as well, with the highest coverage occurring in Beijing and Shanghai, where 88.5% and 79.1% of the population has been fully vaccinated, respectively. In 22 of 31 provincial-level administrative divisions (PLADs), more than 70% of the population was administered at least one dose by August. With the current vaccination capacity, the target of 80% coverage could be achieved by 2021 in 28 PLADs.

**Conclusions:**

Disparities exist in the target population size and vaccination progress across provinces in the mainland of China. China has made great strides in the vaccination speed since roll-out, and could basically achieve the targeted vaccine coverage.

**Graphic Abstract:**

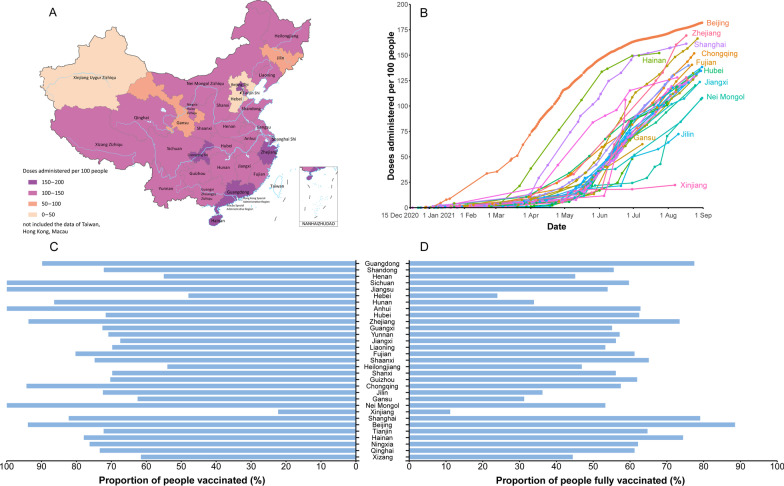

**Supplementary Information:**

The online version contains supplementary material available at 10.1186/s40249-021-00909-1.

## Background

The coronavirus disease 2019 (COVID-19) pandemic still rages on and has caused immeasurable suffering and loss. Owing to effective non-pharmaceutical interventions (NPIs), China achieved outstanding success in fighting the epidemic in early 2020 [1]. Thereafter, since almost the entire population is still susceptible to severe acute respiratory syndrome coronavirus 2 (SARS-CoV-2) [2], China is facing substantial risks of both imported COVID-19 cases and a domestic resurgence in the long run, with over 20 outbreaks of varying scales (from dozens to hundreds of COVID-19 cases reported) occurring frequently in the past year [3]. Relying on stringent NPIs, such as citywide nucleic acid testing and travel restriction [4–6], these local outbreaks were quickly contained.

COVID-19 vaccination, which has been implemented in over 200 countries/regions, is expected to be the long-lasting solution to end the pandemic [7]. In 12 and 21 countries, 70% and 60% of the population has been fully vaccinated respectively as of August 31, 2021. The United States has reached its goal to administer at least one dose of COVID-19 vaccine to 70% of adults on 2 August, 2021, one month behind the schedule. In the mainland of China, the COVID-19 vaccine rollout launched in December 2020 [8]. Great efforts are taken to speed up the vaccination. As of 26 August, 2021, 2.1 billion doses were administered, with 63.4% of the population fully vaccinated [9].

As the most populous country, China aims to vaccinate 80% of its population (1.1 billion) by the end of 2021 [10]. Achieving this goal within such a short period is a major challenge for the Chinese Immunization System. Estimating the target population size is crucial to assist policy-making on COVID-19 vaccine deployment. A previous study quantified the national target population size in China, and estimated the time it would take to achieve target coverage given an assumption of vaccination capacity (i.e., average daily doses administered) [11]. Due to the large variations in population size and vaccination capacity across provinces, the challenge of achieving the above goal varies. Here, we aim to estimate the size of the target population for the COVID-19 vaccination at the provincial level, current vaccination coverage, the relevant demand for vaccination capacity to achieve the aforementioned goal, as well as further identify the gaps between demand and current vaccination capacity in the National Immunization Program (NIP).

## Methods

### Definition of the priority target population for the COVID-19 vaccination program

The current COVID-19 vaccines are licensed for use in people 3 years of age or above. As stated in the Technical Vaccination Recommendations for COVID-19 Vaccines in China [12], individuals with below contraindications are ineligible for vaccination: (1) people who are allergic to any of the components or excipients in the vaccine or substances used in the production process, or who have had allergic reactions after vaccination with similar vaccines; (2) people with a history of severe allergic reaction to any vaccines; (3) people with uncontrolled epilepsy and other serious progressive neurological diseases; (4) people with fever, acute illness, acute exacerbation of a chronic diseases, or uncontrolled severe chronic disease; and (5) pregnant women.

In the mainland of China, COVID-19 vaccination program is initially implemented in adults aged 18 years old and above, and has been extended to individuals aged 12–17 years (6.3% of total population) since late July 2021. It is expected to be further extended to children 3–11 years of age (10.2% of total population) then. To determine the target population, besides aforementioned individuals, children younger than 3 years of age (3.4% of total population) were also included, accounting for vaccines would be approved for use in this age group in the future. The target population was further subdivided into ten priority groups according to the three phased goals, which has been clearly defined and previously endorsed [11, 13]. A brief summary is presented below.

The primary goal is to maintain the essential functions of society. Therefore, essential workers should be vaccinated as the first priority, including, but not limited to, healthcare workers, social security workers, caregivers in social welfare institutions, community workers, personnel producing and supplying daily necessities, and individuals studying/working overseas [11, 13]. These individuals should be considered because of their importance to continuing the functioning of core services and their risk of SARS-CoV-2 infection in an occupational environment.

The secondary goal is to protect the high-risk population, which is more vulnerable to severe clinical illness once infected, including people with underlying conditions and older adults 60 years of age or above [11, 13]. Considering variations in the risk of severe outcomes given SARS-CoV-2 infection across age groups [14, 15], we further categorized the target population into (1) individuals aged 60 or over at least one underlying condition; (2) individuals ≥ 80 years old without underlying conditions; (3) individuals aged 70–79 years old without underlying conditions; (4) individuals aged 60–69 years old without underlying conditions; and (5) individuals < 60 years old with underlying conditions.

The tertiary goal is to contain SARS-CoV-2 transmission, and thus the target population includes other individuals under 60 years of age without underlying conditions, i.e., working-age people (18–59 years), school-aged children (6–17 years) and younger children (0–5 years) [11, 13]. The detailed definition of priority groups is presented in Additional file 1: Table S1.

### Estimating the target population size

To estimate the number of target population, we excluded individuals with aforementioned contraindications to vaccination. That is, serious progressive neurological diseases, and uncontrolled chronic diseases, including idiopathic epilepsy, diabetes mellitus with complications, and uncontrolled hypertension. We extracted the prevalence of individuals with specific diseases in the mainland of China stratified by age and geographic region (Eastern, Central and Western China, details in Additional file 1: Method) from the Global Burden of Diseases, Risk Factors, and Injuries Study in 2019 and the National Health Services Survey in 2013 [16, 17]. Then we estimated the probability of having at least one of these diseases as 1 minus the probability of not having a condition in any of the aforementioned diseases and multiplied the ratio between observed and estimated percentage of individuals with at least one condition reported in the literature [18, 19]. We further multiplied the estimated geographic-region specific probability by the population size of provincial-level administrative divisions (PLADs) in relevant PLADs to produce the number of individuals with any of these conditions.

In addition, pregnant women were excluded. The number of pregnant women is the sum of live births, abortions, still births and fetal deaths, which was obtained from the Chinese Health Statistical Yearbook 2020, Chinese Population & Employment Statistics Yearbook 2020 and previous literature [20, 21]. While the number of people with other contraindications like allergic reaction, acute illness and acute exacerbation of a chronic disease was not available.

The number of essential workers and people studying/working abroad was extracted from statistical yearbooks (i.e., Chinese Economic Census Yearbook 2018, Chinese Civil Affairs Statistical Yearbook 2019, Tabulation data on Population Census), Educational Statistics, the White Paper on China’s National Defense, concise statistics from the Ministry of Commerce, paper books and theses [22–29]. Age-specific population size was obtained from National/Provincial Statistical Yearbooks and the Communiqué of the Seventh National Population Census [30].

People with these underlying conditions are considered to have a high risk of severe outcomes of COVID-19, including respiratory diseases, cardiovascular system diseases, chronic liver diseases, chronic kidney diseases, nervous system diseases, diabetes mellitus, cancers, tuberculosis, the human immunodeficiency virus/acquired immunodeficiency syndrome, sickle cell disorders and a body mass index ≥ 30 (detailed lists of diseases shown in Additional file 1: Table S2). Their size was calculated in the same method as the above contraindications.

Some individuals are classified into more than one group/tier. Under this circumstance, he/she will be sorted into the highest priority when estimating the population size. Additional file 1: Method details the process of removing overlap. Details about the data source are presented in Additional file 1: Table S1.

### Progress of COVID-19 vaccination by PLAD

From December 2020 to 14 March, 2021, the Chinese government periodically reported the total vaccine doses administered at the national level, and the vaccination doses administered have been reported on a daily basis since 23 March, 2021. We collected data on administered doses through official sources (i.e., government websites, health commissions and press conferences) and unofficial sources (i.e., interviews with government officials or persons in charge) by provincial and the Chinese government. Some PLADs report the number of people who have received at least one dose and have been fully vaccinated. For PLADs that did not report this information, we estimated the maximum proportion of the population receiving at least one dose and fully vaccinated.

For 23 of 31 PLADs, the population who has been fully vaccinated was reported. For the rest of provinces, we used a two-dose schedule for inactivated vaccines as a proxy to estimate the coverage of fully vaccinated, accounting for their highest productivity in China (7.7 billion for inactivated vaccines vs 1.2 billion for other vaccines with one-dose or three-dose schedule) (Additional file 1: Table S3).

At the subnational level, the vaccine doses administered are reported irregularly. The COVID-19 vaccination capacity (average daily doses administered) was calculated by the difference in the first and latest vaccine dose data points divided by the time interval. China established a domestic target of vaccinating 80% of the population by 2021’s end [10]. Then we analyzed the expected vaccination doses per day and identified the service gap by province compared to their routine vaccination capacity. Considering that existing vaccines have lower efficacy against Delta variant [31], we set a target of 90% coverage in the sensitivity analysis.

### Routine vaccination capacity by PLAD

The race to roll out COVID-19 vaccines depends on local vaccination capacity. Here, we estimated the provincial-stratified average daily vaccine doses administered before the COVID-19 pandemic, including both free vaccines in the National Immunization Program (NIP vaccines) and self-paid vaccines that have not been introduced into the NIP (non-NIP vaccines). This daily average represents the routine vaccination capacity of the immunization system. The administered doses of NIP vaccines are the product of live births and reported coverage [32]. The coverage of NIP vaccines by PLAD was extracted from government public reports (i.e., Statistical Surveillance Report on Child Development Planning, health statistics data or population health reports) and published literature. The administered doses of non-NIP vaccines in 2014 [33] were used as a proxy for those before the COVID-19 pandemic.

### Data analysis

We carried out statistical evaluation by using the basic statistics and linear correlation. *R*^2^ was calculated to indicate the magnitude of the correlation. Differences with *P*-values < 0.05 were deemed statistically significant. The statistical software R 4.1.0 (Lucent Technologies, Jasmine Mountain, USA) was applied to perform all statistical analyses and plots.

## Results

### Target population size of COVID-19 vaccination by PLAD

**Fig. 1:**
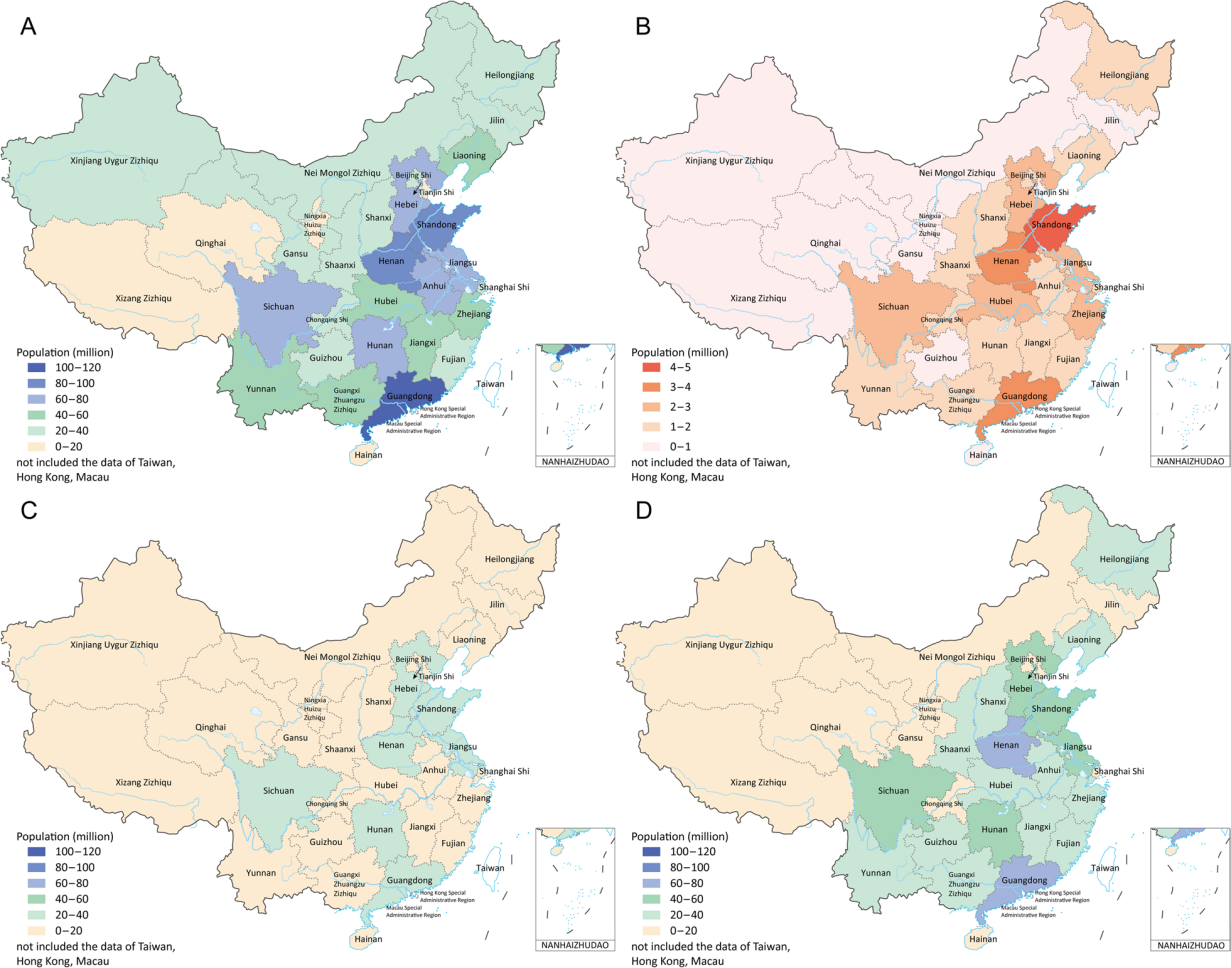
The size of the target population for the COVID-19 vaccination program, stratified by provincial-level administrative division. **A** Overall population. **B** The target population size for maintaining the essential functions of society, including healthcare workers and other critical workers. **C** The target population size for protecting the high-risk population, including people with underlying conditions and older adults. **D** The target population size for containing SARS-CoV-2 transmission, including working-age people, school-aged children and younger children

The national target population is 1.3 billion, and the rest population (100 million) are not eligible for COVID-19 vaccines. From goal 1 to goal 3, a total of 46.9 million, 411.7 million and 864.9 million individuals were included in the vaccination program, respectively. The size of the target population shows large differences among provinces, ranging from 3.4 million to 108.4 million (Fig. 1, Additional file 1: Figure S1 and Table S4). Guangdong province has the largest proportion of the total population. Shandong and Henan provinces rank second and third, accounting for similar shares of 7.1% and 6.9%, respectively. In contrast, Xizang (Tibet), Qinghai, Ningxia and Hainan have a relatively low share of less than 1%.

**Fig. 2:**
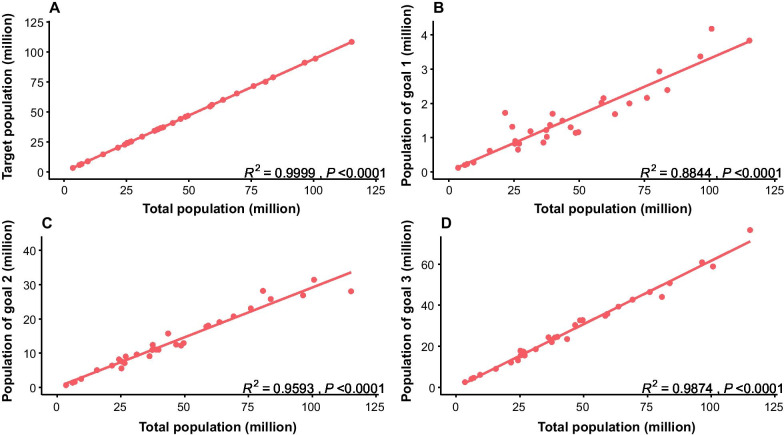
Scatter plots between the size of target population groups and the total population. **A** Target population vs total population. **B** Target population for goal 1 vs total population. **C** Target population for goal 2 vs total population. **D** Target population for goal 3 vs total population

Under the primary goal, Shandong (4.2 million, 8.9%) and Guangdong (3.8 million, 8.2%) provinces have the largest number of essential workers, while Xizang has only 0.3% of healthcare staff and other critical workers in the country. The first eight provinces (Shandong, Guangdong, Henan, Jiangsu, Sichuan, Hebei, Hubei, Zhejiang) together account for 47.7% of the target population of the primary goal. We observed a similar distribution in the target population of the secondary and tertiary goals (Fig. 1 and Additional file 1: Table S5). There was an obvious linear relationship between the size of the target population groups and the total population (Fig. 2).

### Current progress of COVID-19 vaccination and capacity gaps by PLAD

**Fig. 3:**
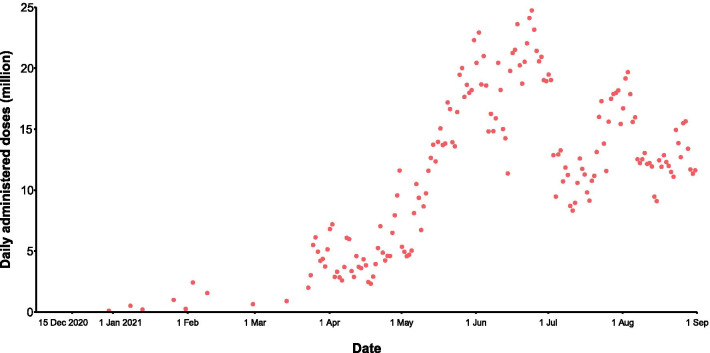
Daily vaccination doses in the mainland of China between 30 November, 2020 and 31 August, 2021

At the initial stage of the COVID-19 vaccination program, the daily doses administered at the national level were very low (< 4 million before 31 March, 2021), and then increased to an average of 4.8 million during April 2021. The daily doses administered have significantly increased since May, and basically stabilized at more than 10 million since 12 May, 2021. The maximum daily doses administered reached 24.7 million on24 June, 2021(Fig. 3).

**Fig. 4:**
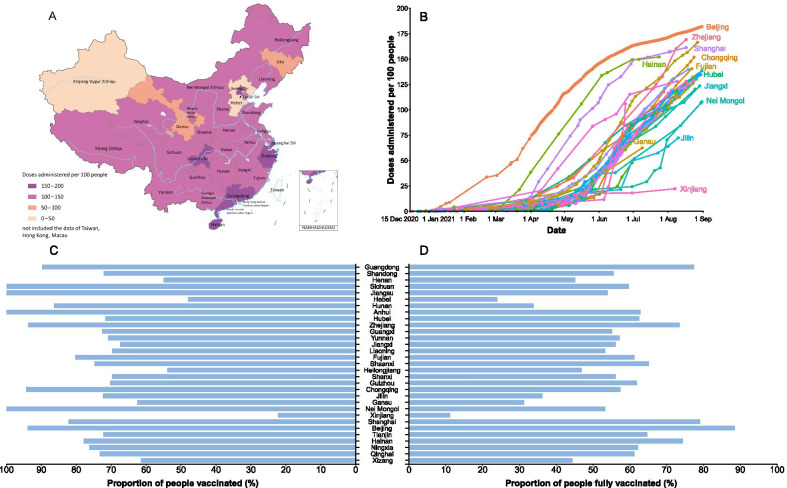
Current cumulative administered doses, vaccination progress and coverage of COVID-19 vaccination program in the mainland of China. **A** Cumulative administered doses as of 31 August, 2021. **B** Time series of administered doses between 15 December, 2020 and 31 August, 2021. **C** The share of the population receiving at least one dose against COVID-19 as of 31 August, 2021. **D** The share of the population fully vaccinated against COVID-19 as of 31 August, 2021

As of 31 August, 2021, a total of two billion doses were administered in the mainland of China, with a median of 48.9 (interquartile range: 23.5–80.0) million doses administered at the provincial level. The five PLADs with the highest doses administered per 100 people (more than 150 doses per 100 people) included Beijing, Zhejiang, Guangdong, Shanghai and Hainan (Fig. 4A, B). The coverage of people administered at least one dose has exceeded 70% in more than two thirds of the PLADs. The coverage of the fully vaccinated population was highest in Beijing, Shanghai, Guangdong, Hainan and Zhejiang, which was over 70%. In 23 of 31 PLADs, more than half of the population has been fully vaccinated (Fig. 4C, D).

**Fig. 5:**
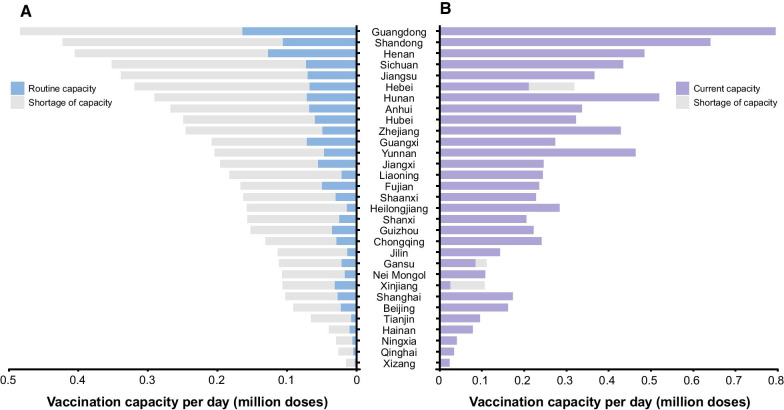
Service capacity gaps to vaccinate 80% of the target population by the end of 2021. **A** The routine daily capacity before the COVID-19 pandemic and service gaps. **B** The current average daily capacity and service gaps

Before the COVID-19 pandemic, the routine vaccination capacity in China was approximately 1.4 million doses per day, equivalent to an average of 1000 doses per million population per day, with the highest vaccination capacity in Guangxi (1439 doses per million population per day) and the lowest vaccination capacity in Heilongjiang (380 doses per million population per day) (Fig. 5A). In more than half of the PLADs, the daily vaccination capacity is between 800 and 1100 doses per million population (Fig. 5A). To achieve a coverage of 80% by the end of 2021 at the provincial level, the average service capacity needs to be increased by 3 to 11 times on the basis of routine capacity (Fig. 5A). By early June 2021, only Beijing, Shanghai, Tianjin and Hainan had achieved the expected daily doses, whereas the other PLADs still faced a daily shortfall of average service capacity, ranging from 5000 doses in Hubei to 0.2 million doses in Hebei province (Additional file 1: Figure S2). However, two months later, only three PLADs still have gaps in their service capacity (Fig. 5B). Even we conducted a targeted coverage of 90% in the sensitivity analysis, 26 PLADs could achieve the goal (Additional file 1: Figure S3).

## Discussion

We quantified the size of the target population groups of COVID-19 vaccination stratified by PLAD in the mainland of China. The size of the target population shows large differences among PLADs, ranging from 3.4 million to 108.4 million. To achieve the goal of vaccinating 80% of the target population by the end of 2021, the COVID-19 vaccination capacity (average daily doses administered) has been highly improved, compared to the routine vaccination capacity before the COVID-19 pandemic. Nevertheless, the speed of vaccine roll-out differs considerably at the provincial level. The highest coverage occurs in Beijing and Shanghai, with 88.5% and 79.1% of the population fully vaccinated, respectively. In 22 of 31 PLADs, more than 70% of the population was administered at least one dose by August 2021. With the current vaccination capacity, the target of 80% coverage could be achieved by 2021 in 28 PLADs.

The Joint Prevention and Control Mechanism of the State Council in China released a three-step strategy for the COVID-19 vaccine rollout on 15 December, 2020 [8]. In the first step, nine groups of essential workers, aged 18–59, were prioritized because of their high risk of occupational exposure. In the second step, vaccination programs would focus on key populations that are vulnerable to severe outcomes once infected, including older adults aged ≥ 60 and people with underlying conditions. In the final step, vaccines are available to other general populations. The vaccination program has been gradually extended to residents aged 18–59 since approximately March 2021 and further extended to people over 60 since 29 March, 2021 [12]. Since late July, the mainland of China has gradually started vaccinations for children aged 12–17 [34]. To vaccinate 80% of the population in 2021, China invested significant efforts into promoting coverage. Estimating the size of target priority groups at the provincial level could support local governments to determine the coverage rate stratified by target groups, and thus to timely determine and adjust vaccination policies and promotional measures.

To accelerate the COVID-19 vaccination and achieve herd immunity as early as possible, a series of measures have been implemented across the mainland of China, including setting up temporary inoculation points, extending the service hours of inoculation points and even opening night vaccination sites, and rolling out mobile vehicles and even offering vaccines door-to-door for those with poor geographical access [35]. Under the joint efforts of central and local governments, the daily vaccination doses have exceeded 17 times the routine capacity (24.7 million vs 1.4 million). Nonetheless, great disparities exist in vaccination progress across PLADs in the early stage of vaccination. For example, more than half of the residents in Beijing and Shanghai have been fully vaccinated by June 2021, whereas the one-shot vaccination proportion was around 30% in most PLADs. The disparities could be largely determined by the national vaccination strategy at the previous stage. Priority is given to areas with higher risks of COVID-19 outbreaks [36], such as Beijing and Shanghai, which are characterized as the largest port cities and the most populous and largest megacities.

In addition to the aforementioned national vaccination strategy, other factors, such as the vaccine supply, varying strengths of local implementation measures and the willingness to receive the COVID-19 vaccination, could also influence vaccination progress. The domestic production capacity is expected to reach 8.9 billion doses this year, thus vaccine supplies would be adequate. The vaccine hesitancy rates varied among PLADs and were above 30% in 20 PLADs [37]. The skyrocketed increase in the number of local populations that received the vaccination after COVID-19 outbreaks occurred in Anhui and Guangdong provinces [38], indicating that stimulating people to receive the vaccination would be crucial to achieving herd immunity as soon as possible.

In this study, we estimate the vaccination capacity needed to reach a coverage target of 80%. Note that it is not a precise estimate of the herd immunity threshold. Instead, it is estimated on the basis of the well-known equation (1 − 1/*R*_0_)/VE [39], where *R*_0_ is the basic reproduction number and VE denotes vaccine efficacy. It ignores heterogeneities that can make these figures biased in specific locations, including social mixing patterns and age-specific susceptibility. Furthermore, the vaccine effectiveness against SARS-CoV-2 variants is lower than that against the prototype [31]. Further modeling studies are needed to determine the exact herd immunity threshold for specific regions.

Several limitations of our study should be mentioned. First, for some contraindications for COVID-19 vaccination, the incidence or prevalence data were not available, including severe allergic reaction, serious progressive neurological diseases, acute illness and acute exacerbation of a chronic diseases. Not excluding individuals with these contraindications may overestimate the size of target population.

Second, when estimating the size of essential workers, we used the latest Chinese Economic Census Yearbook that was updated in 2018. However, the impact of the pandemic on the economic situation might lead to changes in the number of workers in different industries, thus the size might be overestimated or underestimated.

Third, eight PLADs [Sichuan, Anhui, Jiangsu, Nei Mongol (Inner Mongolia), Jilin, Gansu, Hebei and Xinjiang] only announced their cumulative administered doses, without distinguishing the vaccine types. To estimate the number of people who have been fully vaccinated, we assumed a uniform two-dose schedule and simplistically divide the overall doses by two. However, the available data in some PLADs indicated that the proportion of individuals receiving the first dose is much higher than the proportion receiving the second dose, e.g., a difference of 36.8% was observed in Chongqing by 24 August, 2021. Accordingly, we might overestimate the proportion of people fully vaccinated. The exact coverage of fully vaccinated in these PLADs remains to be seen at the provincial level.

Fourth, only Beijing and Shanxi established a system to report daily vaccination status [40, 41]. For other PLADs, we manually collected information through several search engines, which may affect our data integrity. Accordingly, the average service capacity is probably a conservative estimate, due to scarce data points for PLADs, such as Hebei, Jiangsu, Gansu, Xizang and Xinjiang.

Fifth, the routine service capacity was probably a conservative estimate, since we used the administrated doses of non-NIP vaccines in 2014 due to data availability. With the approval and promotion of various non-NIP vaccines (e.g., HPV vaccines, enterovirus A71 vaccines and influenza vaccines) in recent years in the mainland of China, the routine service capacity might be improved more or less.

## Conclusions

Our study quantified the size of priority groups for COVID-19 vaccination stratified by PLADs in the mainland of China. We further provided a landscape for current progress in COVID-19 vaccination and capacity gaps across the mainland of China. The findings show that China has made great strides in the vaccination speed since the start of the vaccine roll-out in late 2020. However, some disparities exist in vaccination progress across PLADs. Based on the current average vaccination capacity, the majority of provinces could reach the vaccine target of 80% by the end of 2021. Further improving vaccination coverage would be crucial to respond to the ongoing COVID-19 pandemic.

## Supplementary Information

**Additional file 1: Method 1.** Three geographic regions of the mainland of China. 2. The process of removing overlaps of the target population groups. **Table S1.** Definitions of priority target groups for the COVID-19 vaccination program. **Table S2.** List of conditions in GBD 2019 with potential to increase the risk of severe COVID-19 illness in China. **Table S3.** The production capacity of the COVID-19 vaccines in China. **Table S4.** The size of each target population for the COVID-19 vaccination program in PLADs. **Table S5.** The size of the target population for the COVID-19 vaccination program by vaccination goals. **Figure S1.** The size of ten priority groups for the COVID-19 vaccination program in PLADs, decreased by the total population. **Figure S2.** Service capacity gaps to vaccinate 80% of the target population by the end of 2021 based on the average capacity by June 2021. **Figure S3.** Service capacity gaps to vaccinate 90% of the target population by the end of 2021.

## Data Availability

All data and data source were provided in details on GitHub at https://github.com/wenzidebanxia/target_population_subnational_China.

## Abbreviations

COVID-19: Coronavirus disease 2019
NPIs: Non-pharmaceutical interventions
SARS-CoV-2: Severe acute respiratory syndrome coronavirus 2
NIP: National Immunization Program
PLADs: Provincial-level administrative divisions
