# Supplementary material for: COVID-19 vaccination program in the mainland of China: a subnational descriptive analysis on target population size and current progress
Source: Infect Dis Poverty. 2021 Oct 15;10:124. doi: 10.1186/s40249-021-00909-1 (PMC8517558; doi:10.1186/s40249-021-00909-1)
Supplement: Supplementary file 1 — Additional file 1: Method 1. Three geographic regions of the mainland of China. 2. The process of removing overlaps of the target population groups. Table S1. Definitions of priority target groups for the COVID-19 vaccination program. Table S2. List of conditions in GBD 2019 with potential to increase the risk of severe COVID-19 illness in China. Table S3. The production capacity of the COVID-19 vaccines in China. Table S4. The size of each target population for the COVID-19 vaccination program in PLADs. Table S5. The size of the target population for the COVID-19 vaccination program by vaccination goals. Figure S1. The size of ten priority groups for the COVID-19 vaccination program in PLADs, decreased by the total population. Figure S2. Service capacity gaps to vaccinate 80% of the target population by the end of 2021 based on the average capacity by June 2021. Figure S3. Service capacity gaps to vaccinate 90% of the target population by the end of 2021. [file 40249_2021_909_MOESM1_ESM.docx]

**Additional file 1**

**COVID-19 vaccination program in the mainland of China: a subnational descriptive analysis on target population size and current progress**

Wen Zheng^1^, Xuemei Yan^1^, Zeyao Zhao^1^, Juan Yang^1,2*^, Hongjie Yu^1,2,3*^

*These authors jointly supervised this work.

*Correspondence: Juan Yang, yangjuan@fudan.edu.cn; Hongjie Yu, yhj@fudan.edu.cn

**Author details**

1. Shanghai Institute of Infectious Disease and Biosecurity, School of Public Health, Fudan University, Shanghai, China

2. Fudan University, Key Laboratory of Public Health Safety, Ministry of Education, Shanghai, China

3. Department of Infectious Diseases, Huashan Hospital, Fudan University, Shanghai, China

**Additional file 1**

[**Method** 3](#_Toc83243220)

[**Table S1.** Definitions of priority target groups for the COVID-19 vaccination program 4](#_Toc83243221)

[**Table S2.** List of conditions in GBD 2019 with potential to increase the risk of severe COVID-19 illness in China 10](#_Toc83243222)

[**Table S3.** The production capacity of the COVID-19 vaccines in China 12](#_Toc83243223)

[**Table S4.** The size of each target population for the COVID-19 vaccination program in PLADs 13](#_Toc83243224)

[**Table S5.** The size of the target population for the COVID-19 vaccination program by vaccination goals 16](#_Toc83243225)

[**Figure S1.** The size of ten priority groups for the COVID-19 vaccination program in PLADs, decreased by the total population. 17](#_Toc83243226)

[**Figure S2.** Service capacity gaps to vaccinate 80% of the target population by the end of 2021 based on the average capacity by June 2021. A: The routine daily capacity before the COVID-19 pandemic and service gaps. B: The average daily capacity as of 10 June and service gaps. 17](#_Toc83243227)

[**Figure S3.** Service capacity gaps to vaccinate 90% of the target population by the end of 2021. A: The routine daily capacity before the COVID-19 pandemic and service gaps. B: The current average daily capacity and service gaps. 18](#_Toc83243228)

[**References** 19](#_Toc83243230)

**Method**

**1.** **Three geographic regions of the mainland of China**

Geographic regions (Eastern, Central and Western China) are divided according to the classification standard of the National Bureau of Statistics [1]. Eastern China includes Beijing, Tianjin, Hebei, Liaoning, Jiangsu, Shanghai, Zhejiang, Fujian, Guangdong, Hainan and Shandong PLADs; Central China includes Jilin, Heilongjiang, Shanxi, Henan, Anhui, Hubei, Hunan and Jiangxi PLADs; Western China includes Nei Mongol, Guangxi, Chongqing, Sichuan, Guizhou, Yunnan, Xizang, Shaanxi, Gansu, Qinghai, Ningxia and Xinjiang PLADs.

**2. The process of removing overlaps of the target population groups**

Before the calculation, there are two assumptions: 1) we assumed that the probability of the population, except pregnant women, with contraindications is equal. When women of childbearing age suffer from serious progressive neurological diseases or uncontrolled chronic diseases, their physical condition is not suitable for pregnancy. Hence, the size of pregnant women with contraindications would be exceedingly small. 2) We assumed that the probability of underlying conditions in the population is equal, and there is no overlap between the population with underlying conditions and with contraindications.

First, the age range of pregnant women is 15-49 years old. We put these people on the first priority to calculate.

Second, the age range of essential workers is 16-79 years old, which means that some of those would be pregnant women. Since the proportion of pregnant women in these groups is unknown, we used the size of essential workers multiply by the proportion of people with contraindications except pregnant women. There are a few pregnant women in this group, thus the size of essential workers would be slightly overestimated.

Third, for individuals with underlying conditions, we excluded pregnant women, essential workers and those with contraindications, and then multiplied by the proportion of people with underlying conditions. For others without underlying conditions, we used the same method to remove overlap, but the multiplier was replaced by the proportion of people without underlying conditions.

**Table S1.** Definitions of priority target groups for the COVID-19 vaccination program

| **Goal** | **Tier** | **Subgroups** | **Target population** | **Definition** | **Data source** |
| --- | --- | --- | --- | --- | --- |
| 1 | 1 | 1 | Healthcare workers | Individuals working at hospitals, community medical institutions, and in the public health system. | The number comes from the China Economic Census Yearbook 2018 [2];  The age structure comes from the tabulation of the 2010 Population Census of the People's Republic of China [3]. |
|  | 2 | 2 | Social security workers | Police officers and the People's Armed Police Force. Even though the People's Liberation Army plays an important role in responding to the epidemic in China, we excluded them here since they are under the direct leadership of the Central Military Commission, which does not involve the allocation of vaccines at the provincial level. | The number of police officers comes from a paper book and master’s thesis [4, 5]; the number of the People's Armed Police Force comes from the White Paper on China’s National Defense [6];  The age structure comes from the tabulation of the 2010 Population Census of the People's Republic of China [3]. |
|  |  |  | Personnel in social welfare institutions | Caregivers in aged organizations and social welfare hospitals. | The number comes from the China Civil Affairs Statistical Yearbook 2019;  The age structure comes from the tabulation of the 2010 Population Census of the People's Republic of China [3]. |
|  |  |  | Community network workers | Individuals working at urban and rural community services. | The number comes from the China Economic Census Yearbook 2018 [2];  The age structure comes from the tabulation of the 2010 Population Census of the People's Republic of China [3]. |
|  |  |  | Personnel in sectors producing and supplying daily necessities like energy, water and transportation | Staff in the agricultural and sideline food processing industry, food manufacturing industry, retailers (i.e., grocery stores, convenience stores, supermarkets, large super markets); personnel in the production and supply of electricity, heat, gas and water industry; and staff in transportation, storage and postal services (i.e., road and water transportation, air transport industry, pipeline transportation, multimodal transport and transport agency industry, handling and warehousing industry, and express industry). | The number comes from the China Economic Census Yearbook 2018 [2];  The age structure comes from the tabulation of the 2010 Population Census of the People's Republic of China [3]. |
|  |  |  | Those studying/working abroad | Individuals studying or working abroad. | The number comes from the Ministry of Education of the People's Republic of China [7] and the Ministry of Commerce of the People's Republic of China [8];  The proportion of individuals studying or working abroad by province comes from paper books [9];  The age structure of those studying abroad comes from a paper book [9]; the age structure of those working abroad comes from the Statistical Yearbook 2020 of PLADs [3, 10-30]. |
| 2 | 3 | 3 | Older adults ≥ 60 years with underlying conditions | Individuals aged 60 or over with underlying conditions, which are on the basis of Clark’s report, and we added a body mass index ≥30. [31] | The number of the permanent population comes from the Communiqué of the Seventh National Population Census, the China Statistical Yearbook 2020 [32] and the Statistical Yearbook 2020 of PLADs [3, 10-30, 33];  The age structure comes from the Communiqué of the Seventh National Population Census and the Statistical Yearbook 2020 of PLADs [3, 10-30, 33];  The prevalence of each underlying condition by age group comes from the Global Burden of Diseases, Risk Factors, and Injuries Study (GBD)[34];  The ratio between observed and estimated percentage of individuals with at least one condition comes from literature[35];  The ratio of the probability of having ≥1 underlying chronic disease in persons among three geographic areas (east, central and west) in China comes from An Analysis Report of National Health Services Survey in China, 2013 [1]; |
|  |  | 4 | Older adults ≥ 80 years without underlying conditions | Individuals aged 80 or over without any underlying condition. | The number of the permanent population comes from the Communiqué of the Seventh National Population Census, the China Statistical Yearbook 2020 [32] and the Statistical Yearbook 2020 of PLADs [3, 10-30, 33];  The age structure comes from the Communiqué of the Seventh National Population Census and the Statistical Yearbook 2020 of PLADs [3, 10-30, 33];  The prevalence of each underlying condition by age group comes from the Global Burden of Diseases, Risk Factors, and Injuries Study (GBD) [34];  The ratio between observed and estimated percentage of individuals with at least one condition comes from literature[35];  The ratio of the probability of having ≥1 underlying chronic disease in persons among three geographic areas (east, central and west) in China comes from An Analysis Report of National Health Services Survey in China, 2013 [1] |
|  | 4 | 5 | Older adults aged 70–79 years without underlying conditions | Individuals aged 70–79 without any underlying condition. | The number of the permanent population comes from the Communiqué of the Seventh National Population Census, the China Statistical Yearbook 2020 [32] and the Statistical Yearbook 2020 of PLADs [3, 10-30, 33];  The age structure comes from the Communiqué of the Seventh National Population Census and the Statistical Yearbook 2020 of PLADs [3, 10-30, 33];  The age structure comes from the Statistical Yearbook 2020 of PLADs [3, 10-30];  The prevalence of each underlying condition by age group comes from the Global Burden of Diseases, Risk Factors, and Injuries Study (GBD) [34];  The ratio of the probability of having ≥1 underlying chronic disease in persons among three geographic areas (east, central and west) in China comes from An Analysis Report of National Health Services Survey in China, 2013 [1] |
|  |  | 6 | Older adults aged 60–69 years without underlying conditions | Individuals aged 60–69 without any underlying condition. | The number of the permanent population comes from the Communiqué of the Seventh National Population Census, the China Statistical Yearbook 2020 [32] and the Statistical Yearbook 2020 of PLADs [3, 10-30, 33];  The age structure comes from the Communiqué of the Seventh National Population Census and the Statistical Yearbook 2020 of PLADs [3, 10-30, 33];  The prevalence of each underlying condition by age group comes from the Global Burden of Diseases, Risk Factors, and Injuries Study (GBD) [34];  The ratio of the probability of having ≥1 underlying chronic disease in persons among three geographic areas (east, central and west) in China comes from An Analysis Report of National Health Services Survey in China, 2013 [1] |
|  |  | 7 | Individuals < 60 years with underlying conditions | Individuals aged 0–59 with underlying conditions. | The number of the permanent population comes from the Communiqué of the Seventh National Population Census, the China Statistical Yearbook 2020 [32] and the Statistical Yearbook 2020 of PLADs [3, 10-30, 33];  The age structure comes from the Communiqué of the Seventh National Population Census and the Statistical Yearbook 2020 of PLADs [3, 10-30, 33];  The prevalence of each underlying condition by age group comes from the Global Burden of Diseases, Risk Factors, and Injuries Study (GBD) [34];  The ratio between observed and estimated percentage of individuals with at least one condition comes from literature[35];  The ratio of the probability of having ≥1 underlying chronic disease in persons among three geographic areas (east, central and west) in China comes from An Analysis Report of National Health Services Survey in China, 2013 [1] |
| 3 | 5 | 8 | Adults aged 18–59 years without underlying conditions | Individuals aged 18–59 without any underlying condition. | The number of permanent populations comes from the China Statistical Yearbook 2020 [32];  The age structure comes from the Statistical Yearbook 2020 of PLADs and the China Population & Employment Statistics Yearbook 2020 [36]. |
|  | 6 | 9 | School-aged children | School students aged 6–17. | The number of permanent populations comes from the China Statistical Yearbook 2020 [32];  The age structure comes from the Statistical Yearbook 2020 of PLADs and the China Population & Employment Statistics Yearbook 2020 [36]. |
|  |  | 10 | Younger children | Individuals aged 0–5. | The number of permanent populations comes from the China Statistical Yearbook 2020 [32];  The age structure comes from the Statistical Yearbook 2020 of PLADs and the China Population & Employment Statistics Yearbook 2020 [36]. |

**Table S2.** List of conditions in GBD 2019 with potential to increase the risk of severe COVID-19 illness in China

| **No.** | **Category** | **Causes included in the Global Burden of Disease Study (GBD 2019)** |
| --- | --- | --- |
| 1 | HIV/AIDS | HIV/AIDS - Drug-susceptible Tuberculosis; HIV/AIDS - Multidrug-resistant Tuberculosis without extensive drug resistance; HIV/AIDS - Extensively drug-resistant Tuberculosis; HIV/AIDS resulting in other diseases |
| 2 | Tuberculosis | Drug-susceptible tuberculosis; Multidrug-resistant tuberculosis without extensive drug resistance; Extensively drug-resistant tuberculosis |
| 3 | Cancers with direct immune suppression | Hodgkin lymphoma; Non-Hodgkin lymphoma; Multiple myeloma; Acute lymphoid leukemia; Chronic lymphoid leukemia; Acute myeloid leukemia; Chronic myeloid leukemia; Other leukemia; Other malignant neoplasms; Myelodysplastic, myeloproliferative, and other hematopoietic neoplasms |
| 4 | Cancers with possible immune suppression (from treatment therapy) | Lip and oral cavity cancer; Nasopharynx cancer; Other pharynx cancer; Esophageal cancer; Stomach cancer; Colon and rectum cancer; Liver cancer due to hepatitis B; Liver cancer due to  hepatitis C; Liver cancer due to alcohol use; Liver cancer due to NASH; Liver cancer due to other causes; Gallbladder and biliary tract cancer; Pancreatic cancer; Larynx cancer; Tracheal, bronchus, and lung cancer; Malignant skin melanoma; Breast cancer; Cervical cancer; Uterine cancer; Ovarian cancer; Prostate cancer; Testicular cancer; Kidney cancer; Bladder cancer; Brain and nervous system cancer; Thyroid cancer; Mesothelioma |
| 5 | Cardiovascular disease | Rheumatic heart disease; Ischemic heart disease; Ischemic stroke; Intracerebral haemorrhage; Subarachnoid haemorrhage; Non-rheumatic calcific aortic valve disease; Non-rheumatic degenerative mitral valve disease; Other non-rheumatic valve diseases; Myocarditis; Alcoholic cardiomyopathy; Other cardiomyopathy; Atrial fibrillation and flutter; Endocarditis; Other cardiovascular and circulatory diseases; Congenital heart anomalies; Lower extremity peripheral arterial disease |
| 6 | Chronic respiratory disease | Chronic obstructive pulmonary disease; Silicosis; Asbestosis; Coal workers pneumoconiosis; Other pneumoconiosis; Asthma; Interstitial lung disease and pulmonary sarcoidosis |
| 7 | Chronic liver disease | Cirrhosis and other chronic liver diseases due to hepatitis B; Cirrhosis and other chronic liver diseases due to hepatitis C; Cirrhosis and other chronic liver diseases due to alcohol use; Cirrhosis and other chronic liver diseases due to other causes |
| 8 | Diabetes without complications | Diabetes mellitus type 1; Diabetes mellitus type 2 |
| 9 | Chronic kidney disease | Chronic kidney disease due to diabetes mellitus type 1; Chronic kidney disease due to diabetes mellitus type 2; Chronic kidney disease due to glomerulonephritis; Chronic kidney disease due to other and unspecified causes |
| 10 | Chronic neurological disorders | Alzheimer's disease and other dementias; Parkinson's disease; Multiple sclerosis; Motor neuron disease; Other neurological disorders; Idiopathic developmental intellectual disability; Down syndrome; Neural tube defects |
| 11 | Controlled hypertension | Hypertensive heart disease; Chronic kidney disease due to hypertension |
| 12 | Obesity | BMI≥30 |
| 13 | Sickle cell disorders | Sickle cell disorders |

**Table S3.** The production capacity of the COVID-19 vaccines in China

| Vaccine type | Manufacturer | Vaccination schedule | Production capacity |
| --- | --- | --- | --- |
| Inactivated | Sinovac | 2 doses | 2 billion [37] |
| Inactivated | Sinopharm | 2 doses | 5 billion [38] |
| Inactivated | Shenzhen Kangtai Biological Products | 2 doses | 200–600 million [39] |
| Inactivated | Chinese Academy of Medical Sciences | 2 doses | 500 million–1 billion [40] |
| Adenovirus vector | CanSino | 1 dose | 700 million [41] |
| Recombinant subunit | Anhui Zhifei Longcom Biopharmaceutical products | 3 doses | 500 million [42] |

**Table S4.** The size of each target population for the COVID-19 vaccination program in PLADs

|  |  | Target population (thousand persons, %) | | | | | | | | | |  |
| --- | --- | --- | --- | --- | --- | --- | --- | --- | --- | --- | --- | --- |
|  | **All** | Healthcare workers | Other critical workers | Older adults ≥ 60 years with underlying conditions | Older adults ≥ 80 years without underlying conditions | Older adults aged 70–79 years without underlying conditions | Older adults aged 60–69 years without underlying conditions | Individuals < 60 years with underlying conditions | Adults aged 18–59 years without underlying conditions | School-aged children | Younger children ≤ 5 years | |
| Subtotal | 1323509 (100.0) | 10387 (100.0) | 36505 (100.0) | 105119 (100.0) | 10718 (100.0) | 38066 (100.0) | 79067 (100.0) | 178766 (100.0) | 588791 (100.0) | 185642 (100.0) | 90449 (100.0) | |
| PLADs |  |  |  |  |  |  |  |  |  |  |  | |
| Beijing | 20222 (1.5) | 322 (3.1) | 1403 (3.8) | 1558 (1.5) | 180 (1.7) | 408 (1.1) | 1120 (1.4) | 3117 (1.7) | 9808 (1.7) | 1358 (0.7) | 947 (1.0) | |
| Tianjin | 14722 (1.1) | 124 (1.2) | 494 (1.4) | 1424 (1.4) | 127 (1.2) | 445 (1.2) | 993 (1.3) | 2063 (1.2) | 6684 (1.1) | 1530 (0.8) | 839 (0.9) | |
| Hebei | 71631 (5.4) | 465 (4.5) | 1696 (4.6) | 6347 (6.0) | 523 (4.9) | 2056 (5.4) | 4392 (5.6) | 9750 (5.5) | 29694 (5.0) | 9976 (5.4) | 6731 (7.4) | |
| Shanxi | 35322 (2.7) | 256 (2.5) | 966 (2.6) | 2792 (2.7) | 245 (2.3) | 1054 (2.8) | 2194 (2.8) | 4665 (2.6) | 15793 (2.7) | 5322 (2.9) | 2033 (2.2) | |
| Nei Mongol | 24074 (1.8) | 191 (1.8) | 709 (1.9) | 1817 (1.7) | 165 (1.5) | 825 (2.2) | 1723 (2.2) | 3023 (1.7) | 11479 (1.9) | 2874 (1.5) | 1268 (1.4) | |
| Liaoning | 40775 (3.1) | 353 (3.4) | 1144 (3.1) | 4699 (4.5) | 400 (3.7) | 1475 (3.9) | 3330 (4.2) | 5846 (3.3) | 17935 (3.0) | 3951 (2.1) | 1642 (1.8) | |
| Jilin | 25441 (1.9) | 201 (1.9) | 626 (1.7) | 2447 (2.3) | 217 (2.0) | 890 (2.3) | 1984 (2.5) | 3500 (2.0) | 11891 (2.0) | 2565 (1.4) | 1119 (1.2) | |
| Heilongjiang | 35529 (2.7) | 247 (2.4) | 779 (2.1) | 3110 (3.0) | 244 (2.3) | 1123 (3.0) | 2639 (3.3) | 5327 (3.0) | 17643 (3.0) | 3112 (1.7) | 1305 (1.4) | |
| Shanghai | 22748 (1.7) | 241 (2.3) | 1079 (3.0) | 2439 (2.3) | 296 (2.8) | 729 (1.9) | 1530 (1.9) | 3195 (1.8) | 10504 (1.8) | 1711 (0.9) | 1024 (1.1) | |
| Jiangsu | 75166 (5.7) | 632 (6.1) | 2300 (6.3) | 8201 (7.8) | 767 (7.2) | 2456 (6.5) | 5745 (7.3) | 11023 (6.2) | 31911 (5.4) | 8447 (4.6) | 3685 (4.1) | |
| Zhejiang | 54598 (4.1) | 527 (5.1) | 1493 (4.1) | 4678 (4.4) | 487 (4.5) | 1526 (4.0) | 2939 (3.7) | 8109 (4.5) | 25698 (4.4) | 6253 (3.4) | 2888 (3.2) | |
| Anhui | 60062 (4.5) | 387 (3.7) | 1302 (3.6) | 4738 (4.5) | 524 (4.9) | 1786 (4.7) | 3367 (4.3) | 8657 (4.8) | 26438 (4.5) | 8746 (4.7) | 4117 (4.6) | |
| Fujian | 37304 (2.8) | 266 (2.6) | 1431 (3.9) | 2378 (2.3) | 188 (1.8) | 601 (1.6) | 1972 (2.5) | 5847 (3.3) | 17498 (3.0) | 4321 (2.3) | 2802 (3.1) | |
| Jiangxi | 44190 (3.3) | 285 (2.7) | 1019 (2.8) | 2895 (2.8) | 331 (3.1) | 1004 (2.6) | 2220 (2.8) | 6068 (3.4) | 19954 (3.4) | 6901 (3.7) | 3513 (3.9) | |
| Shandong | 94424 (7.1) | 741 (7.1) | 3437 (9.4) | 8963 (8.5) | 875 (8.2) | 2908 (7.6) | 5784 (7.3) | 12897 (7.2) | 38786 (6.6) | 12660 (6.8) | 7375 (8.2) | |
| Henan | 91113 (6.9) | 678 (6.5) | 2693 (7.4) | 6325 (6.0) | 568 (5.3) | 1998 (5.2) | 5585 (7.1) | 12394 (6.9) | 38632 (6.6) | 15246 (8.2) | 6995 (7.7) | |
| Hubei | 55970 (4.2) | 457 (4.4) | 1697 (4.6) | 4819 (4.6) | 449 (4.2) | 1797 (4.7) | 3710 (4.7) | 7284 (4.1) | 24460 (4.2) | 7422 (4.0) | 3876 (4.3) | |
| Hunan | 65435 (4.9) | 468 (4.5) | 1532 (4.2) | 5563 (5.3) | 603 (5.6) | 2229 (5.9) | 3825 (4.8) | 8514 (4.8) | 28097 (4.8) | 9210 (5.0) | 5396 (6.0) | |
| Guangdong | 108428 (8.2) | 824 (7.9) | 3009 (8.2) | 6044 (5.7) | 628 (5.9) | 1916 (5.0) | 3937 (5.0) | 15530 (8.7) | 51043 (8.7) | 17979 (9.7) | 7519 (8.3) | |
| Guangxi | 46865 (3.5) | 344 (3.3) | 817 (2.2) | 3089 (2.9) | 446 (4.2) | 1389 (3.6) | 2505 (3.2) | 5510 (3.1) | 20016 (3.4) | 8120 (4.4) | 4627 (5.1) | |
| Hainan | 8892 (0.7) | 73 (0.7) | 205 (0.6) | 593 (0.6) | 64 (0.6) | 201 (0.5) | 359 (0.5) | 1258 (0.7) | 3969 (0.7) | 1436 (0.8) | 735 (0.8) | |
| Chongqing | 29439 (2.2) | 236 (2.3) | 951 (2.6) | 2560 (2.4) | 348 (3.2) | 1171 (3.1) | 2052 (2.6) | 3512 (2.0) | 12740 (2.2) | 4139 (2.2) | 1732 (1.9) | |
| Sichuan | 78934 (6.0) | 622 (6.0) | 1768 (4.8) | 6811 (6.5) | 937 (8.7) | 3105 (8.2) | 5464 (6.9) | 9486 (5.3) | 34925 (5.9) | 11194 (6.0) | 4622 (5.1) | |
| Guizhou | 34367 (2.6) | 273 (2.6) | 586 (1.6) | 2061 (2.0) | 225 (2.1) | 983 (2.6) | 1738 (2.2) | 4129 (2.3) | 14730 (2.5) | 6874 (3.7) | 2768 (3.1) | |
| Yunnan | 46007 (3.5) | 330 (3.2) | 811 (2.2) | 2677 (2.5) | 329 (3.1) | 1218 (3.2) | 2286 (2.9) | 5717 (3.2) | 21765 (3.7) | 7597 (4.1) | 3276 (3.6) | |
| Xizang | 3375 (0.3) | 17 (0.2) | 104 (0.3) | 107 (0.1) | 11 (0.1) | 44 (0.1) | 105 (0.1) | 410 (0.2) | 1606 (0.3) | 643 (0.3) | 328 (0.4) | |
| Shaanxi | 36697 (2.8) | 334 (3.2) | 1036 (2.8) | 2706 (2.6) | 263 (2.5) | 1208 (3.2) | 2525 (3.2) | 4344 (2.4) | 16383 (2.8) | 5448 (2.9) | 2450 (2.7) | |
| Gansu | 25147 (1.9) | 175 (1.7) | 476 (1.3) | 1646 (1.6) | 133 (1.2) | 790 (2.1) | 1492 (1.9) | 3044 (1.7) | 11359 (1.9) | 4326 (2.3) | 1707 (1.9) | |
| Qinghai | 5794 (0.4) | 52 (0.5) | 149 (0.4) | 267 (0.3) | 21 (0.2) | 124 (0.3) | 250 (0.3) | 724 (0.4) | 2753 (0.5) | 1007 (0.5) | 446 (0.5) | |
| Ningxia | 6571 (0.5) | 55 (0.5) | 177 (0.5) | 341 (0.3) | 30 (0.3) | 157 (0.4) | 317 (0.4) | 808 (0.5) | 3053 (0.5) | 1133 (0.6) | 501 (0.6) | |
| Xinjiang | 24264 (1.8) | 212 (2.0) | 613 (1.7) | 1025 (1.0) | 94 (0.9) | 453 (1.2) | 982 (1.2) | 3016 (1.7) | 11545 (2.0) | 4142 (2.2) | 2182 (2.4) | |

**Table S5.** The size of the target population for the COVID-19 vaccination program by vaccination goals

|  |  | Vaccination program goals (thousand persons, %) | | |
| --- | --- | --- | --- | --- |
|  | **All** | Maintaining essential social functions | Reducing severe outcomes | Reducing illness/transmission |
| Subtotal | 1323509 (100.0) | 46892 (100.0) | 411735 (100.0) | 864881 (100.0) |
| PLADs |  |  |  |  |
| Beijing | 20222 (1.5) | 1725(3.7) | 6383(1.6) | 12114(1.4) |
| Tianjin | 14722 (1.1) | 618(1.3) | 5052(1.2) | 9053(1.0) |
| Hebei | 71631 (5.4) | 2161(4.6) | 23068(5.6) | 46402(5.4) |
| Shanxi | 35322 (2.7) | 1222(2.6) | 10951(2.7) | 23149(2.7) |
| Nei Mongol | 24074 (1.8) | 901(1.9) | 7553(1.8) | 15620(1.8) |
| Liaoning | 40775 (3.1) | 1497(3.2) | 15750(3.8) | 23528(2.7) |
| Jilin | 25441 (1.9) | 828(1.8) | 9038(2.2) | 15575(1.8) |
| Heilongjiang | 35529 (2.7) | 1025(2.2) | 12444(3.0) | 22060(2.6) |
| Shanghai | 22748 (1.7) | 1320(2.8) | 8189(2.0) | 13239(1.5) |
| Jiangsu | 75166 (5.7) | 2932(6.3) | 28191(6.8) | 44043(5.1) |
| Zhejiang | 54598 (4.1) | 2019(4.3) | 17739(4.3) | 34839(4.0) |
| Anhui | 60062 (4.5) | 1689(3.6) | 19071(4.6) | 39302(4.5) |
| Fujian | 37304 (2.8) | 1698(3.6) | 10986(2.7) | 24621(2.8) |
| Jiangxi | 44190 (3.3) | 1304(2.8) | 12518(3.0) | 30367(3.5) |
| Shandong | 94424 (7.1) | 4178(8.9) | 31426(7.6) | 58820(6.8) |
| Henan | 91113 (6.9) | 3371(7.2) | 26869(6.5) | 60873(7.0) |
| Hubei | 55970 (4.2) | 2154(4.6) | 18058(4.4) | 35758(4.1) |
| Hunan | 65435 (4.9) | 1999(4.3) | 20734(5) | 42702(4.9) |
| Guangdong | 108428 (8.2) | 3833(8.2) | 28054(6.8) | 76541(8.8) |
| Guangxi | 46865 (3.5) | 1161(2.5) | 12940(3.1) | 32763(3.8) |
| Hainan | 8892 (0.7) | 278(0.6) | 2475(0.6) | 6140(0.7) |
| Chongqing | 29439 (2.2) | 1187(2.5) | 9642(2.3) | 18610(2.2) |
| Sichuan | 78934 (6.0) | 2391(5.1) | 25803(6.3) | 50741(5.9) |
| Guizhou | 34367 (2.6) | 859(1.8) | 9136(2.2) | 24372(2.8) |
| Yunnan | 46007 (3.5) | 1142(2.4) | 12227(3) | 32638(3.8) |
| Xizang | 3375 (0.3) | 121(0.3) | 677(0.2) | 2577(0.3) |
| Shaanxi | 36697 (2.8) | 1370(2.9) | 11046(2.7) | 24281(2.8) |
| Gansu | 25147 (1.9) | 651(1.4) | 7104(1.7) | 17391(2.0) |
| Qinghai | 5794 (0.4) | 201(0.4) | 1387(0.3) | 4206(0.5) |
| Ningxia | 6571 (0.5) | 232(0.5) | 1653(0.4) | 4687(0.5) |
| Xinjiang | 24264 (1.8) | 825(1.8) | 5570(1.4) | 17870(2.1) |

**Figure S1.** The size of ten priority groups for the COVID-19 vaccination program in PLADs, decreased by the total population.


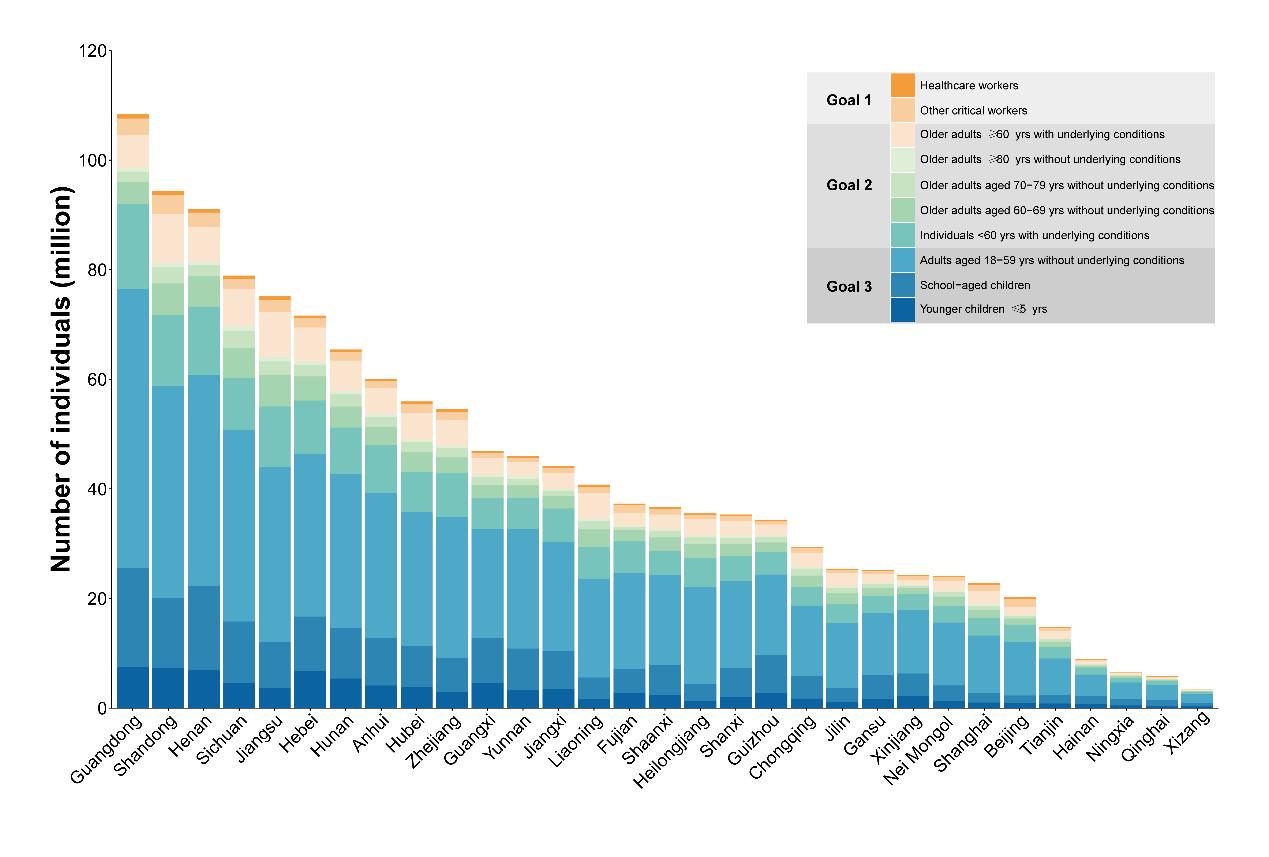


**Figure S2.** Service capacity gaps to vaccinate 80% of the target population by the end of 2021 based on the average capacity by June 2021. A: The routine daily capacity before the COVID-19 pandemic and service gaps. B: The average daily capacity as of June 10 and service gaps.


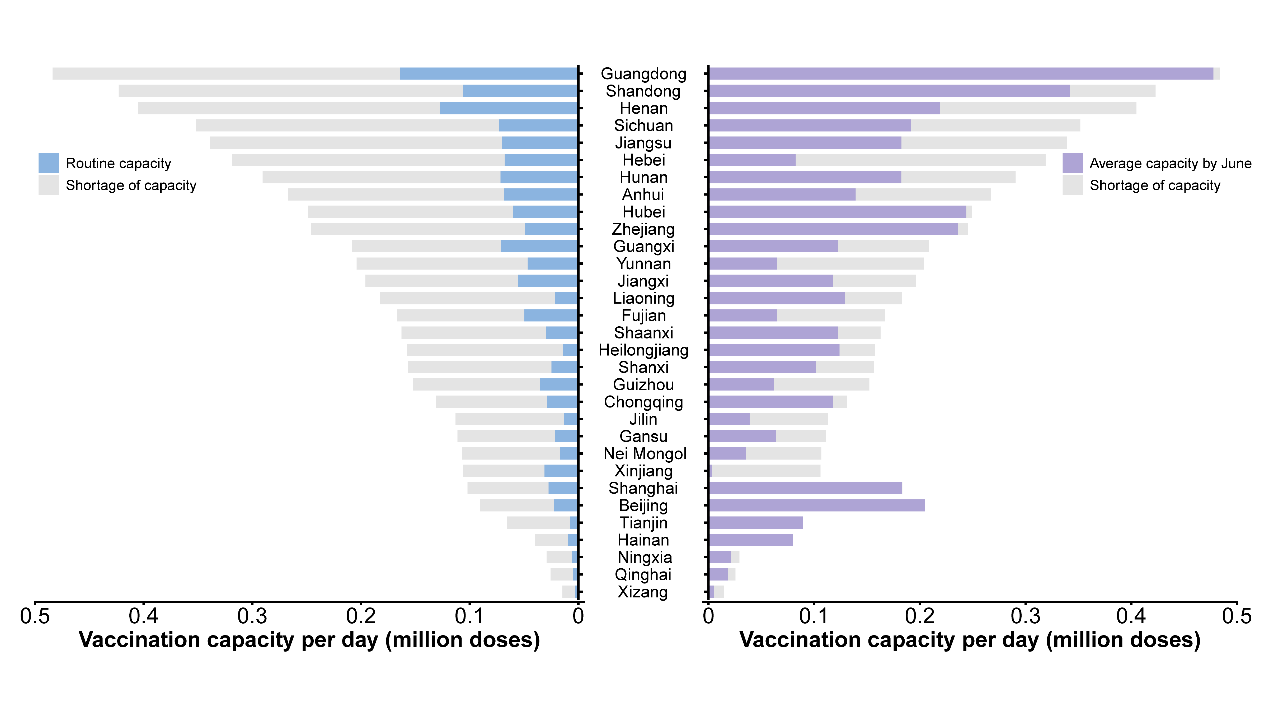


**Figure S3.** Service capacity gaps to vaccinate 90% of the target population by the end of 2021. A: The routine daily capacity before the COVID-19 pandemic and service gaps. B: The current average daily capacity and service gaps.


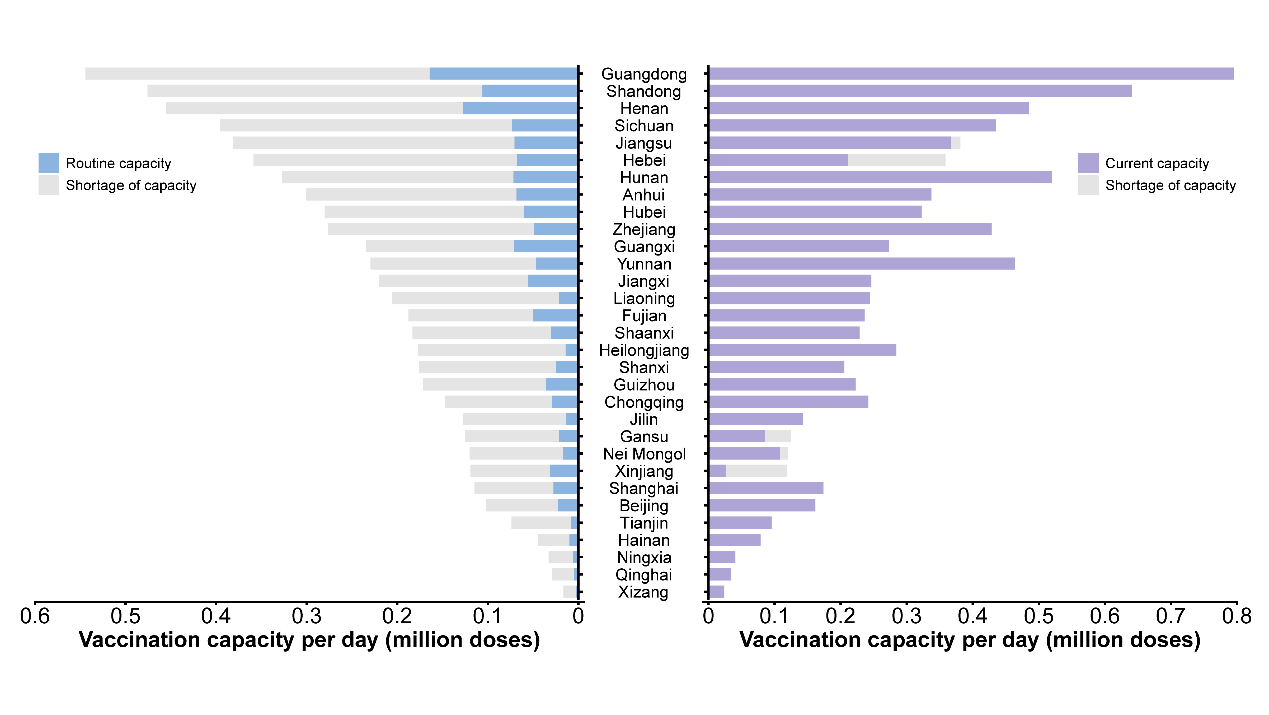


**References**

1. Center for Health Statistics and Information. An Analysis Report of National Health Services Survey in China, 2013. <http://www.nhc.gov.cn/ewebeditor/uploadfile/2016/10/20161026163512679.pdf>. Accessed on 7 April 2021.

2. The Fourth National Economic Census. China Economic Census Yearbook 2018. <http://www.stats.gov.cn/tjsj/pcsj/jjpc/4jp/zk/indexch.htm>. Accessed on 3 August 2021.

3. National Bureau of Statistics. Tabulation the 2010 Population Census of the People's Republic of China. <http://www.stats.gov.cn/tjsj/pcsj/rkpc/6rp/indexch.htm>. Accessed on 30 Nov 2020.

4. Li Q: Research on the Optimal Allocation of Police Human Resources Based on Big Data. People’s Public Security University of China; 2020.

5. Fan P: Social transformation and state coercion. Beijing: China Social Sciences Press; 2017.

6. Ministry of National Defense of the People's Republic of China. White Paper on "China's National Defense In 2006". <http://www.mod.gov.cn/regulatory/2011-01/06/content_4617808_4.htm>. Accessed on 30 Nov 2020.

7. Ministry of Education of the People's Republic of China. The considerations of college entrance examination delay; How to arrange the resumption of classes? <http://www.moe.gov.cn/jyb_xwfb/s5147/202004/t20200401_437149.html>. Accessed on 30 Nov 2020.

8. Ministry of Commerce. PRC. Concise statistics on China's foreign labor service cooperation business from January to October 2020. <http://hzs.mofcom.gov.cn/article/date/202011/20201103018497.shtml>. Accessed on 30 Nov 2020.

9. Service Center for Overseas Study, Ministry of Education. Blue book of return and employment of Chinese overseas students. Beijing: Yanshi Press in China; 2015.

10. Qinghai Bureau of Statistics. Qinghai statistical Yearbook 2020. <http://tjj.qinghai.gov.cn/nj/2020/indexch.htm>. Accessed on 3 June 2021.

11. Gansu Bureau of Statistics. Gansu development Yearbook 2020. <http://tjj.gansu.gov.cn/tjnj/2020/indexch.htm>. Accessed on 3 June 2021.

12. Shaanxi Bureau of Statistics. Shaanxi statistical Yearbook 2020. <http://219.144.222.224:81/tjj/upload/n2020/indexch.htm>. Accessed on 3 June 2021.

13. Guizhou Bureau of Statistics. Guizhou statistical Yearbook 2020. <http://202.98.195.171:81/tj/2020/zk/indexch.htm>. Accessed on 3 June 2021.

14. Chongqing Bureau of Statistics. Chongqing statistical Yearbook 2020. <http://tjj.cq.gov.cn/zwgk_233/tjnj/2020/indexch.htm>. Accessed on 3 June 2021.

15. Hainan Bureau of Statistics. Hainan statistical Yearbook 2020. <http://stats.hainan.gov.cn/tjj/tjsu/ndsj/2020/index.html>. Accessed on 3 June 2021.

16. Guangxi Bureau of Statistics. Guangxi statistical Yearbook 2020. <http://tjj.gxzf.gov.cn//tjsj/tjnj/material/tjnj20200415/2020/zk/indexch.htm>. Accessed on 3 June 2021.

17. Guangdong Bureau of Statistics. Guangdong statistical Yearbook 2020. <http://stats.gd.gov.cn/gdtjnj/content/post_3098041.html>. Accessed on 3 June 2021.

18. Shandong Bureau of Statistics. Shandong statistical Yearbook 2020. <http://tjj.shandong.gov.cn/tjnj/nj2020/zk/indexch.htm>. Accessed on 3 June 2021.

19. Jiangxi Bureau of Statistics. Jiangxi statistical Yearbook 2020. <http://tjj.jiangxi.gov.cn/resource/nj/2020CD/indexch.htm>. Accessed on 3 June 2021.

20. Fujian Bureau of Statistics. Fujian statistical Yearbook 2020. <http://tjj.fujian.gov.cn/tongjinianjian/dz2020/index.htm>. Accessed on 3 June 2021.

21. Anhui Bureau of Statistics. Anhui statistical Yearbook 2020. <http://tjj.ah.gov.cn/oldfiles/tjj/tjjweb/tjnj/2020/cn.html>. Accessed on 3 June 2021.

22. Jiangsu Bureau of Statistics. Jiangsu statistical Yearbook 2020. <http://tj.jiangsu.gov.cn/2020/nj03/nj0307.htm>. Accessed on 3 June 2021.

23. Heilongjiang Bureau of Statistics. Heilongjiang statistical Yearbook 2020. <http://tjj.hlj.gov.cn/app/tongjnj/2020/zk/indexch.htm>. Accessed on 3 June 2021.

24. Liaoning Bureau of Statistics. Liaoning statistical Yearbook 2020. <http://tjj.ln.gov.cn/tjsj/sjcx/ndsj/otherpages/2020/2020/indexch.htm>. Accessed on 3 June 2021.

25. Nei Mongol Bureau of Statistics. Nei Mongol Statistical Yearbook 2020. <http://tj.nmg.gov.cn/files_pub/content/PAGEPACK/b85658190a3644f8b192e45f5221f2fa/indexch.htm>. Accessed on 2 June 2021.

26. Tianjin Bureau of Statistics. Tianjin statistical Yearbook 2020. <http://stats.tj.gov.cn/nianjian/2020nj/zk/indexch.htm>. Accessed on 2 June 2021.

27. Beijing Bureau of Statistics. Beijing statistical Yearbook 2020. <http://nj.tjj.beijing.gov.cn/nj/main/2020-tjnj/zk/indexch.htm>. Accessed on 2 June 2021.

28. Shanxi Bureau of Statistics. Shanxi Statistical Yearbook 2020. <http://tjj.shanxi.gov.cn/tjsj/tjnj/nj2020/zk/indexch.htm>. Accessed on 3 June 2021.

29. Henan Bureau of Statistics. Henan statistical Yearbook 2019. <http://oss.henan.gov.cn/sbgt-wztipt/attachment/hntjj/hntj/lib/tjnj/2019/zk/indexch.htm>. Accessed on 3 June 2021.

30. Hebei Bureau of Statistics. Hebei Economic Yearbook 2019. <http://tjj.hebei.gov.cn/res/nj2019/indexch.htm>. Accessed on 3 August 2021.

31. Yang J, Zheng W, Shi H, Yan X, Dong K, You Q et al. Who should be prioritized for COVID-19 vaccination in China? A descriptive study. BMC Med. 2021; 191:45.

32. National Bureau of Statistics. China Statistical Yearbook 2020. <http://www.stats.gov.cn/tjsj/ndsj/2020/indexch.htm>. Accessed on 7 April 2021.

33. National Bureau of Statistics of China. Communiqué of the Seventh National Population Census. <http://www.stats.gov.cn/english/StatisticalCommuniqu/>. Accessed on 31 August 2021.

34. Global Health Data Exchange. Global Burden of Diseases, Risk Factors, and Injuries Study (GBD). <http://ghdx.healthdata.org/gbd-results-tool>. Accessed on 7 April 2021.

35. Wang HH, Wang JJ, Wong SY, Wong MC, Li FJ, Wang PX et al. Epidemiology of multimorbidity in China and implications for the healthcare system: cross-sectional survey among 162,464 community household residents in southern China. BMC Med. 2014; 12:188.

36. National Bureau of Statistics. China Population & Employment Statistics Yearbook 2020. <https://navi.cnki.net/KNavi/YearbookDetail?pcode=CYFD&pykm=YZGRL&bh>=. Accessed on 3 June 2021.

37. China Sinovac says it reached two billion doses annual capacity for COVID-19 vaccine. <https://www.reuters.com/article/us-health-coronavirus-vaccine-sinovac-idUSKBN2BP07G>. Accessed on 24 August 2021.

38. Sinopharm's annual production capacity of COVID-19 vaccine reaches 5 billion doses. <https://baijiahao.baidu.com/s?id=1709255688902042355&wfr=spider&for=pc>. Accessed on 30 August 2021.

39. Kangtai biological COVID-19 vaccine is approved for emergency use, designed annual production capacity of 200 million doses which can be increased to 600 million doses. <https://baijiahao.baidu.com/s?id=1700375683105781562&wfr=spider&for=pc>. Accessed on 24 August 2021.

40. COVID-19 inactivated vaccine developed by Chinese Academy of Medical Sciences was first released for emergency use. <https://www.imbcams.ac.cn/Item/24586.aspx>. Accessed on 14 August 2021.

41. CanSino Bio's COVID-19 vaccine production base will help boost China's production capacity of single dose vaccine. <https://baijiahao.baidu.com/s?id=1698018408150218416&wfr=spider&for=pc>. Accessed on 24 August 2021.

42. Zhifei Biotech's COVID-19 vaccine accelerates production, with a daily output of more than 1 million doses. <https://baijiahao.baidu.com/s?id=1700161504516615796&wfr=spider&for=pc>. Accessed on 24 August 2021.
